# Design of a Signal-Amplified Aptamer-Based Lateral Flow Test Strip for the Rapid Detection of Ochratoxin A in Red Wine

**DOI:** 10.3390/foods11111598

**Published:** 2022-05-28

**Authors:** Yinyin Liu, Dan Liu, Shuangshuang Cui, Can Li, Ziguang Yun, Jian Zhang, Fengxia Sun

**Affiliations:** School of Food Science and Technology, Shihezi University, Shihezi 832000, China; liuyinyin98@163.com (Y.L.); dliu8011@163.com (D.L.); alexandra9982022@163.com (S.C.); li1821632098@163.com (C.L.); yydwy2022@163.com (Z.Y.); sfx315518@163.com (J.Z.)

**Keywords:** ochratoxin A, signal amplification, lateral flow test strip, aptamer, red wine

## Abstract

In order to improve the weak optical performance of gold nanoparticles and realize the signal amplification of lateral flow chromatography test strips, individual gold nanoparticles (AuNPs) were aggregated into gold nanoparticle aggregates through functional groups around polyamidoamine (PAMAM) dendrimers. A signal-amplified aptamer-based lateral flow chromatography test strip was constructed for the rapid determination of ochratoxin A (OTA). Under optimal conditions, the visual detection limit of this test strip was 0.4 ng mL^−1^ and the semi-quantitative limit of detection (LOD) was 0.04 ng mL^−1^. Compared with other traditional aptamer lateral flow chromatography test strips, its sensitivity was improved about five times. The whole test could be completed within 15 min. The aptamer-based strip was applied to the detection of OTA in red wine; the average recoveries ranged from 93% to 105.8% with the relative standard deviation (RSD) varying from 3% to 8%, indicating that the test strip may be a potentially effective tool for the on-site detection of OTA.

## 1. Introduction

Ochratoxin A (OTA) is a secondary metabolite produced by several toxin-producing strains of *Aspergillus* and *Penicillium*. It is widely found in coffee, beer, red wine, grape juice, corn, wheat, vegetables, meat and meat products [1,2]. After being absorbed by the human body, it develops strong hepatotoxicity and nephrotoxicity as well as teratogenic, carcinogenic, mutagenic and immunosuppressive effects [3]. The International Agency for Research on Cancer has classified it as a class 2B carcinogen [4]. The European Commission has set limits on OTA in cereal-based food intended for young children (0.5 μg kg^−1^), grape juice and wine (2 μg kg^−1^), processed cereals (3 μg kg^−1^), raw grains (5 μg kg^−1^), roasted coffee (5 μg kg^−1^) and dried fruits (10 μg kg^−1^) [5,6]. At present, the instrumental analysis methods used to detect OTA include high-performance liquid chromatography (HPLC), liquid chromatography–mass spectrometry (LC–MS) and gas chromatography–mass spectrometry (GC–MS) [7]. Although these methods are sensitive and accurate, they require professionals to operate large and expensive instruments and the process of sample preparation is complex. Although an enzyme-linked immunosorbent assay (ELISA) [8] and an immunochromatographic assay (ICA) [9] do not need large instruments, they rely on specific antibodies. The period of antibody preparation is laborious and it is difficult and costly for small molecule compounds such as OTA.

In recent years, aptamers have attracted greater attention due to their advantages of a strong specificity, a wide range of target substances, easy modification, a strong stability at room temperature and a low cost to detect OTA [10,11]. Nekrasov et al. developed an aptasensor on the basis of a graphene field-effect transistor array for OTA detection [12]. The LOD was 1.4 pM in phosphate-buffered saline (PBS) and 1 pM in wine. However, for the on-site detection and large-scale screening of mycotoxins, lateral flow chromatography strips may be a better choice. In previous reports, the visual LOD of this strip for qualitative detection was 1 ng mL^−1^ whereas the LOD for semi-quantitative detection was as low as 0.18 ng mL^−1^ [13,14]. However, the weak optical properties of colloidal gold and the low binding rate of biomolecules can lead to a low sensitivity of traditional gold-labeled lateral flow chromatography strips, which means it is difficult to meet the requirements of a rapid analysis of trace hazards in food and other media [15]. Therefore, how to improve the detection sensitivity of lateral flow chromatography strips has become a research focus.

Polyamidoamine (PAMAM) is composed of alkyl amine- and tertiary amine-branched chains, which have a high structural flexibility as well as hydrophilicity, good biocompatibility and chemical stability [16]. It is widely used as a sensing medium in electrochemical sensors for signal amplification because it has a large number of terminal functional groups on its periphery, which can greatly increase the amount of various immobilized biomolecules or nanomaterials [17]. Shen et al. designed gold nanoparticle aggregates based on PAMAM to enhance the sensitivity of antibody-based strips for macromolecules; a result almost 20-fold lower than the traditional method using individual gold nanoparticles was achieved [18]. Similarly, we previously developed a signal-enhanced strip biosensor for small molecule detection based on PAMAM binding with antibodies [19]. This signal-enhanced method exhibited at least a 50-fold improvement of the sensitivity. It is worth noting that the antibody-based and aptamer-based strip biosensors were based on different recognition and strip design models. Therefore, it is of great importance and interest to develop signal-amplified aptamer-based strip biosensors.

In this study, we present a novel signal-amplified aptamer-based strip for the visual and ultrasensitive detection of OTA. PAMAM was used to aggregate gold nanoparticles into gold nanoclusters to enhance the chromogenic effect; an excellent sensitivity was achieved under optimized conditions, providing a potential and universal signal amplification technique for the determination of trace pollutants. In addition, we applied the signal-amplified aptamer-based strip sensor to the detection of OTA in red wine samples. The interference of the red wine matrix was eliminated by the sample preparation.

## 2. Materials and Methods

### 2.1. Reagents and Materials

Ochratoxin A (OTA), aflatoxin B1 (AFB1), zearalenone (ZEN), fumonisin B1 (FB1), deoxynivalenol (DON) and polyamidoamine (PAMAM, G4.0) dendrimers as well as HAuCl_4_·3H_2_O were purchased from Sigma-Aldrich (St. Louis, MO, USA). Streptavidin, sucrose, Tween-20, trisodium citrate and deoxyadenosine triphosphate (dATP) were purchased from Sangon Biotech Co., Ltd. (Shanghai, China). Tris (2-carboxyethyl) phosphine (TCEP), sodium lauryl sulphate (SDS), ovalbumin (OVA), bovine serum albumin (BSA) and polyethylene glycol 20,000 (PEG 20,000) were provided by Shanghai Aladdin Biochemical Technology Co., Ltd. (Shanghai, China). Sodium chloride (NaCl), potassium carbonate (K_2_CO_3_), sodium hydrogen phosphate (Na_2_HPO_4_), monobasic potassium (KH_2_PO_4_) and potassium chloride (KCl) were all purchased from Sinopharm Chemical Reagent Co., Ltd. (Shanghai, China). An anti-OTA aptamer and other ssDNA probes were synthesized by Sangon Biotech Co., Ltd. The sequence of the OTA aptamer was 5′-GAT CGG GTG TGG GTG GCG TAA AGG GAG CAT CGG ACA AAA AAA AAA AAA AAA AAA-SH-3′. The T line probe was 5′-Biotin-TGT CCG ATG CTC CCT TTA CGC CAC CCA CAC CCG ATC-3′ (probe 1) and the C line probe was 5′-Biotin-TTT TTT TTT TTT TTT TTT-3′ (probe 2). The glass fiber membrane (sample pad), polyester fiber membrane (conjugate pad), nitrocellulose membrane (NC membrane) and absorbent pad were purchased from Shanghai Kinbio Tech Co., Ltd. (Shanghai, China). All the solutions were prepared with ultrapure water (resistivity: 18.2 MΩ·cm).

### 2.2. Synthesis of AuNPs

AuNPs (10 ± 2 nm) were synthesized by using tannic acid and sodium citrate as reducing agents. Briefly, 1 mL of 1% HAuCl_4_ was added into a flask containing 79 mL of sterile distilled water. Secondly, 4 mL of 1% sodium citrate solution, 0.1 mL of 1% tannic acid and 0.1 mL of K_2_CO_3_ (2.5 × 10^−2^ mol L^−1^) solution were added into another flask containing 15.8 mL of sterile distilled water. The above two solutions were then kept at 60 °C for 30 min. Finally, the two solutions were mixed together under high-speed stirring and kept at 60 °C until the color turned wine red and no longer changed. The solution was stored at 4 °C.

### 2.3. Preparation of the PAMAM–AuNP–Aptamer Bioconjugates

First, 5 mL of the AuNP solution was added to K_2_CO_3_ (0.1 mol L^−1^) to adjust the pH to 8.0–9.0. PAMAM (5 × 10^−5^ mol L^−1^) was then added and reacted for 5 h at room temperature. After centrifugation (12,000 rpm, 20 min), it was resuspended in 1 mL of sterile distilled water and stored at 4 °C. The aptamer (1 × 10^−4^ mol L^−1^) was then added to 1 mL PAMAM–AuNP and reacted at 4 °C for 12 h. The NaCl solution with a final concentration of 5 × 10^−4^ mol L^−1^ was then added and the reaction continued for another 24 h. The mixture was purified by centrifugation at 10,000 rpm for 20 min. After two washes with PBS, the PAMAM–AuNP–aptamer bioconjugates were resuspended in the conjugate resuspension solution (0.01 mol L^−1^ PBS, 0.5% PEG, 5% sucrose, 1% OVA and 0.25% Tween-20) and stored at 4 °C.

### 2.4. Fabrication of the Signal-Amplified Aptamer Strip

The lateral flow chromatography test strip was composed of a glass fiber membrane (sample pad), a polyester fiber membrane (conjugate pad), an NC membrane and an absorbent pad. Primarily, the sample pad was soaked in PBS containing 2% sucrose and 0.25% Tween-20 for 30 min and dried at 60 °C for 2 h. The conjugate pad was soaked in PBS containing 4% sucrose, 1% OVA and 0.25% Tween-20 for 24 h and dried at 60 °C for 2 h. Streptavidin was dissolved in PBS (0.01 mol L^−1^, pH 7.4) and 40 μL of streptavidin (1 mg mL^−1^) was added to 40 μL of the probe solution (15 μM). The mixture was then added to 40 μL of PBS and incubated at 4 °C for 2 h. Subsequently, the prepared probe 1 solution and probe 2 solution were sprayed onto the NC membrane as the test line (T line) and the control line (C line), respectively, for the subsequent analysis. Finally, the processed sample pad and conjugate pad as well as the NC membrane and absorbent pad were glued to a rubber board in a certain order. The pasted strip was cut into small strips with a width of 3 mm by a slitting machine. The lateral flow chromatography test strips were then stored in a self-sealed bag.

### 2.5. Test Procedure

In brief, standard solutions with different OTA concentrations were prepared by spiking OTA into the buffer solution (10% methanol in 0.01 M PBS). In a typical test, 80 μL of OTA standard solution with a specific concentration was added to the sample pad. Due to the capillary action, the solution could flow into the conjugate pad that contained the PAMAM–AuNP–aptamer conjugate. After 10 min, a change in color of the test zone on the strip was easily observed with the naked eye. The color intensities of the T and C lines were read with the use of Image J software. The quantitative analysis was carried out by their signal-to-noise ratio (S/N).

### 2.6. Detection of OTA in Red Wine Samples

A total of 30 μL of OTA standard solution (0.01 mg mL^−1^) was diluted with 2.97 mL of red wine; the final concentration of OTA in the sample reached 100 ng mL^−1^. Subsequently, 20 mL of methanol, 0.5 g of sodium acetate and 2 g of anhydrous magnesium sulfate were fully mixed with the spiked red wine by shaking for 3 min. After standing for 30 min, the mixture was centrifuged at 8000 rpm for 10 min and the supernatant was collected. This step was repeated twice. The obtained supernatant was merged and then evaporated to a near dry condition at 40 °C under a vacuum. The obtained sample solution was redissolved in an aqueous solution of methanol. After the sample solution was filtered with a 0.22 μm microporous membrane, the volume was increased to 3 mL. Finally, a certain volume of the sample solution was diluted to different OTA concentrations by running a buffer solution (5× saline sodium citrate (SSC) containing 1% BSA + 0.2% Tween-20 + 0.01% SDS) for the detection.

## 3. Results and Discussion

### 3.1. Principle of the Signal-Amplified Aptamer-Based Strip

**Figure 1:**
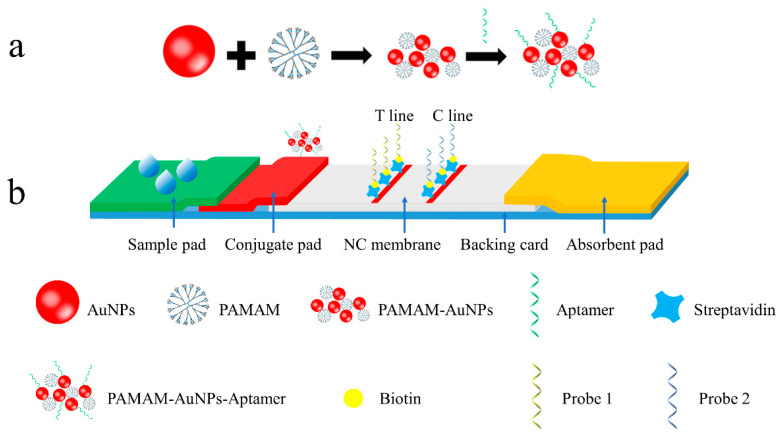
(**a**) The schematic illustration for the preparation of PAMAM–AuNP–aptamer aggregates; (**b**) the sensing principle of the signal-amplified lateral flow strip.

The principle of the signal-amplified strip based on PAMAM and an aptamer was that the target OTA competed with the captured probe 1 to bind to the PAMAM–AuNP–aptamer conjugate, as shown in Figure 1. When the strip was immersed in the sample solution, the sample solution could migrate in the direction of the absorbent pad under the action of the capillary power. The PAMAM–AuNP–aptamer conjugate on the conjugate pad was redissolved and then was mixed with the sample solution to migrate on the strip. When OTA was present in the sample solution, OTA and the PAMAM–AuNP–aptamer conjugates combined, the PAMAM–AuNP aptamer captured by probe 1 on the T line decreased and the color became weaker. As the OTA concentration increased, the T line disappeared whereas the C line remained. In other words, the degree of color rendering on the T line was inversely proportional to the concentration of OTA. This was a competitive lateral flow assay for OTA detection. Regardless of whether there was OTA in the sample or not, the C line was colored to ensure the validity of the test strip.

Taking advantage of the special structure of PAMAM, multiple AuNPs were fixed around it to form gold nanoclusters. When the PAMAM–AuNP–aptamer conjugate reached the T line by migration, probe 1 on the T line captured more AuNPs than the single AuNPs of the traditional lateral flow strip, which improved the color intensity on the T line.

### 3.2. Characterization of AuNPs, PAMAM–AuNPs and PAMAM–AuNP–Aptamers

**Figure 2:**
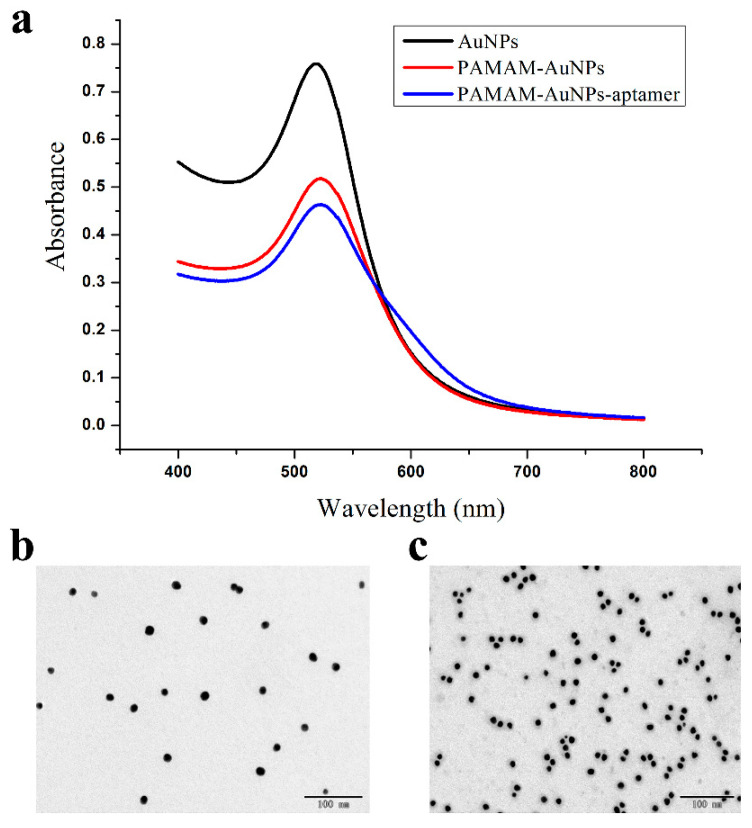
(**a**) The UV–Vis spectra of AuNPs, PAMAM–AuNPs and PAMAM–AuNP–aptamer aggregates; (**b**) the TEM image of AuNPs; (**c**) the TEM image of PAMAM–AuNPs.

Due to the characteristics of localized surface plasmon resonance (LSPR) [20], AuNPs are used as indicators for lateral flow chromatography test strips. They have a strong ultraviolet absorption in the visible region, so the maximum absorption wavelength of 518 nm (Figure 2a) can be detected by a UV–Vis spectrophotometer. As shown in Figure 2a, when the AuNPs were modified with PAMAM and aptamers, the maximum absorption wavelength was red-shifted and the maximum absorption wavelengths were 522 nm and 523 nm, respectively. The result indicated the formation of PAMAM–AuNPs and PAMAM–AuNP–aptamer conjugates. At the same time, AuNPs and PAMAM–AuNPs were characterized by a transmission electron microscope (TEM). As seen in the figure, the dispersion of AuNPs was good and the particle size was uniform (Figure 2b). When adding PAMAM to the AuNP solution, AuNPs combined with PAMAM under the action of static electricity and multiple AuNPs were stacked together and there were holes around them (Figure 2c).

### 3.3. Optimization of the Experimental Conditions

**Figure 3:**
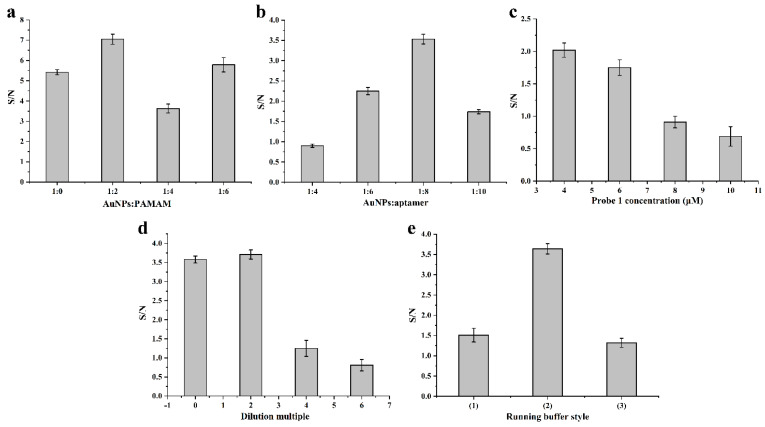
Effect of the ratio of AuNPs to PAMAM (**a**), the ratio of AuNPs to aptamers (**b**), probe 1 concentration (**c**), dilution multiple (**d**) and running buffer style (**e**) on the S/N of the test strip. S represents the peak area of the test zone and N represents the peak area of the control zone.

In order to obtain the best performance, the ratio of AuNPs to PAMAM, the ratio of AuNPs to aptamers, the probe 1 concentration, the dilution multiple of PAMAM–AuNP–aptamer conjugates and the type of running buffer were systematically studied. The results are shown in Figure 3. First of all, the amount of PAMAM added affected the color-rendering degree of the T line and C line. As shown in Figure 3a, when the ratio of AuNPs to PAMAM was 1:2, the S/N value was the highest. The ratio of PAMAM–AuNPs to aptamers was then optimized. When the ratio was 1:6, the conjugate was stable, but the signal strength was low. It can be observed from Figure 3b that the S/N value was the largest when the ratio was 1:8. Moreover, the optimal ratio was 1:8 under the premise of ensuring the stability and sensitivity of the PAMAM–AuNP–aptamer conjugate. If the concentration of the PAMAM–AuNP–aptamer conjugate was too large, there was a strong background color, which affected the sensitivity of the test strip. Therefore, the conjugate was diluted twice. At the same time, the S/N value was also the highest, as shown in Figure 3d.

In addition, if the concentration of probe 1 on the T line was too high, the test strip was prone to false negatives. In the case of ensuring a clear color development, an appropriate concentration was selected. Figure 3c presents that the S/N value decreased as the concentration of probe 1 on the T line decreased. When the concentration of probe 1 was 10 μM, the S/N value was the highest. There were three running buffers: (1) PBST: 0.01 mol L^−1^ PBS + 1% Tween-20; (2) 5× SSC containing 1% BSA + 0.2% Tween-20 + 0.01% SDS; and (3) 200 mM Tris-HCl + 100 mM KCl + 100 mM (NH_4_)_2_SO_4_ + 100 mM MgSO_4_ containing 1% polyvinyl pyrrolidone, 0.6% sucrose and 8% PEG 20,000. Figure 3e shows that the running buffer of 5 × SSC containing 1% BSA, 0.2% Tween-20 and 0.01% SDS had a higher S/N value. The conditions after optimization were as follows: the ratio of AuNPs to PAMAM was 1:2; the ratio of AuNPs to aptamers was 1:8; probe 1 concentration was 10 μM; the dilution multiple of the PAMAM–AuNP–aptamer conjugates was twice; and the type of running buffer was buffer (2).

### 3.4. Analytical Performance of the Strip

**Figure 4:**
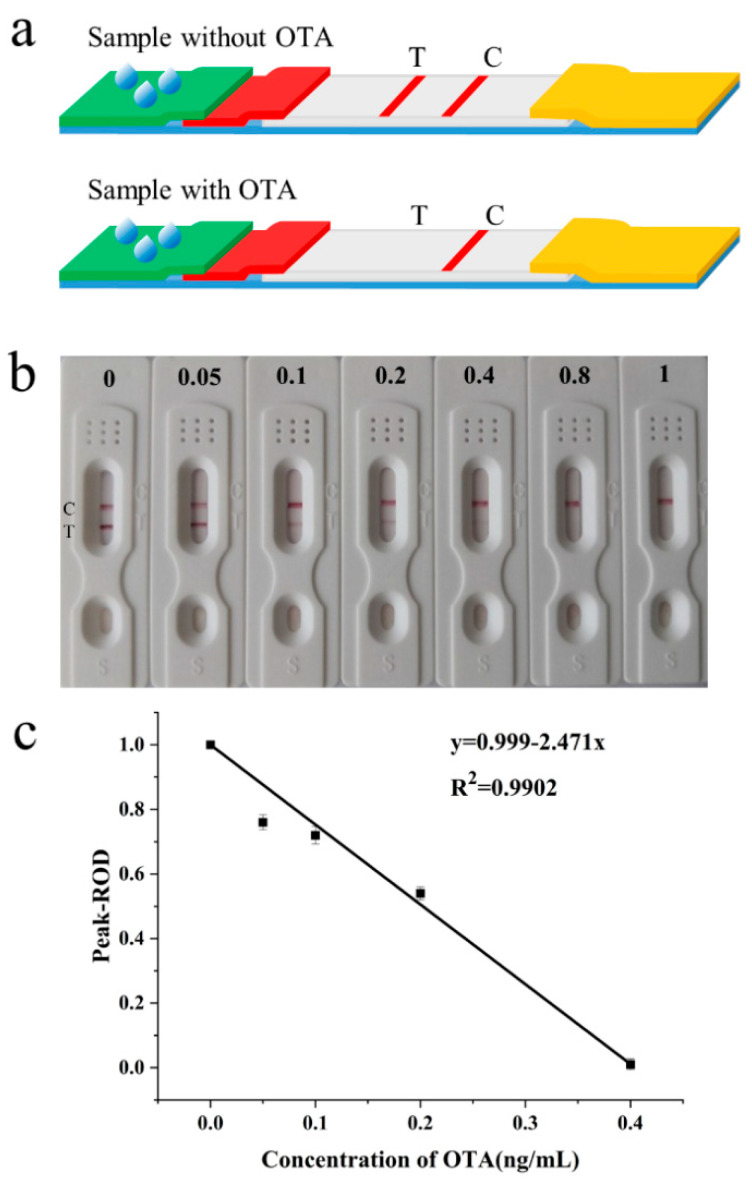
(**a**) The expected test results of the sample without and with OTA; (**b**) sensitivity test of the test strip; (**c**) calibration curve for the detection of OTA via Image J software.

The expected test results in the presence and absence of OTA are shown in Figure 4a. Based on the above optimized conditions, this test strip was used to detect OTA at concentrations of 0.05 ng mL^−1^, 0.1 ng mL^−1^, 0.2 ng mL^−1^, 0.4 ng mL^−1^, 0.8 ng mL^−1^ and 1 ng mL^−1^, respectively. Each concentration was measured three times and the results are shown in Figure 4b. It can be seen from the figure that with an increase in the OTA concentration, the color degree of the T line became weaker. When the OTA concentration was 0.4 ng mL^−1^, the T line became far weaker; when the OTA concentration reached 0.8 ng mL^−1^, the T line disappeared. Therefore, according to the definition of the visual LOD [21], 0.4 ng mL^−1^ could be regarded as the visual LOD of the aptamer-based test strip. The optical density of the T line was measured by Image J software and then the peak area-relative optical density (Peak-ROD) of OTA in the solution was calculated by comparing the peak optical density value of the T line obtained with that detected in PBS. According to the definition of a semi-quantitative LOD, it could be calculated from the calibration curve by Origin 2020, as seen in Figure 4c, that the semi-quantitative LOD of the test strip was 0.04 ng mL^−1^. Compared with a conventional antibody-based test strip, the sensitivity of this study was 25 times higher [22] and about 5 times higher than that of aptamer-based test strips [13,14].

**Figure 5:**
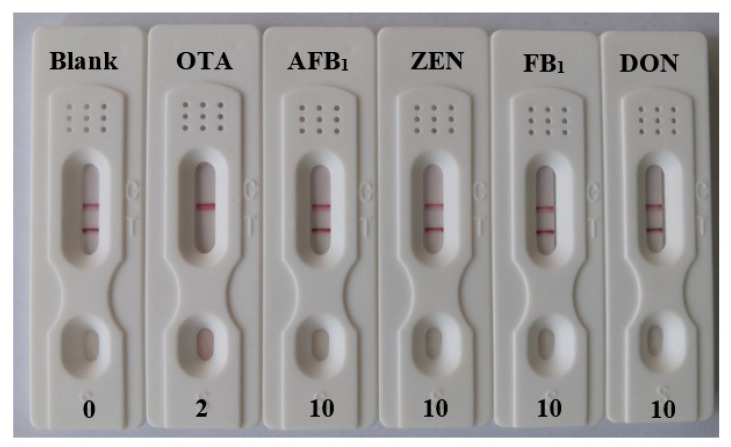
Detection results of the specificity confirmation of the test strip.

In order to test the specificity of the test strip, four types of common mycotoxins with a similar structure to OTA were chosen for the detection. Under optimal conditions, the test strips were inserted into multiple microwells containing 100 μL OTA, AFB1, ZEN, FB1 and DON, respectively, in which OTA was 2 ng mL^−1^ and other mycotoxins were 10 ng mL^−1^. The test results are shown in Figure 5. It could be seen that when the target was OTA only, the T line disappeared, indicating that the strip had an excellent selectivity and specificity for OTA.

In order to verify the stability, test strips of same batch were sealed in a packbag containing a desiccant and stored at 4 °C for 7 days, 14 days, 21 days and 30 days, respectively. The OTA concentration of 0.1 ng mL^−1^ was then detected. Compared with the newly prepared test strips, the T line colors of the test strips stored for 7 days, 14 days and 21 days were similar. The test strips stored for 30 days showed a slightly weaker color, but the effect of color loss in the detection process was negligible. Hence, the test strips worked well after being sealed and stored at 4 °C for 30 days, showing a good stability.

### 3.5. Practical Application of the Test Strip

In order to evaluate the practicality of the test strip, red wine was purchased from the local supermarket. A standard solution of OTA with a known concentration was added to prepare OTA-contaminated samples of 0.05, 0.1, 0.4, 0.5 and 1 ng mL^−1^. After being processed, the spiked samples were tested with the test strips. The results are shown in Table 1, which illustrates that the average recovery rates changed from 93% to 105.8% with the RSD ranging from 3% to 8%, demonstrating that the proposed method had an acceptable accuracy, precision and practicality for quantitative OTA detection in wine samples. In addition, there is an overview of the comparison with other previous reported methods in Table 2, which reveals that our developed method showed a lower LOD. This could be used for the rapid and sensitive analysis of OTA.

## 4. Conclusions

In this study, a highly sensitive aptamer-based lateral flow test strip was constructed using PAMAM–AuNPs instead of a single AuNP. Multiple AuNPs gathered together through the amino groups around PAMAM and then immobilized on the T line, thereby improving the sensitivity of the test strip. Under optimum conditions, the visual LOD was 0.4 ng mL^−1^ and the semi-quantitative LOD was 0.04 ng mL^−1^. The influence of the red wine matrix on the test was eliminated by a sample pretreatment. Moreover, the test strip had a good selectivity and specificity and could be used for the analysis of OTA in red wine. The recoveries and the RSD were 93–105.8% and 3–8%, respectively. Hence, the aptamer-based lateral flow chromatography test strip could be a promising tool for the on-site screening and detection of OTA in more than red wine.

## Figures and Tables

**Table 1:** Results of OTA detection in red wine samples.

^a^ Group	Add (ng mL^−1^)	Found (ng mL^−1^)	^b^ Recovery (%)	^b^ RSD (%)
1	0.05	0.050 ± 0.004	103	8
2	0.1	0.095 ± 0.003	96	3
3	0.4	0.42 ± 0.02	106	4
4	0.5	0.49 ± 0.02	101	3
5	1	0.93 ± 0.04	93	4

^a^ Each group had three samples; ^b^ *n* = 3.

**Table 2:** Comparison of the method proposed in this study with other reported methods for OTA detection using lateral flow strips.

Labels	Signal Output	Recognition Probe	LOD (ng mL^−1^)	Assay Time (min)	Reference
AuNPs	Colorimetry	Antibody	1	5	[22]
AuNPs/AgNPs	Colorimetry	Antibody	0.9	10	[23]
Quantum dots	Fluorescence	Antibody	0.07	15	[24]
Quantum dot nanobeads	Fluorescence	Antibody	0.085	15	[25]
Magneto–gold nanohybrid	Colorimetry	Antibody	0.094	5	[26]
AuNPs	Colorimetry	Aptamer	0.18	10	[14]
AuNPs	Colorimetry	Aptamer	1	15	[13]
Cy5	Fluorescence	Aptamer	0.4	10	[7]
AuNPs	Colorimetry	Aptamer	0.04	15	This study

## Data Availability

The data presented in this study are available on request from the corresponding author.

## References

[1] Liu C., Guo Y., Luo F., Rao P., Fu C., Wang S. (2016). Homogeneous Electrochemical Method for Ochratoxin A Determination Based on Target Triggered Aptamer Hairpin Switch and Exonuclease III-Assisted Recycling Amplification. Food Anal. Methods.

[2] Mehri F., Esfahani M., Heshmati A., Jenabi E., Khazaei S. (2022). The prevalence of ochratoxin A in dried grapes and grape-derived products: A systematic review and meta-analysis. Toxin Rev..

[3] Heshmati A., Mehri F., Nili-Ahmadabadi A., Khaneghah A.M. (2021). The fate of ochratoxin A during grape vinegar production. Int. J. Environ. Anal. Chem..

[4] Badie Bostan H., Danesh N.M., Karimi G., Ramezani M., Mousavi Shaegh S.A., Youssefi K., Charbgoo F., Abnous K., Taghdisi S.M. (2017). Ultrasensitive detection of ochratoxin A using aptasensors. Biosens. Bioelectron..

[5] Jiang C., Lan L., Yao Y., Zhao F., Ping J. (2018). Recent progress in application of nanomaterial-enabled biosensors for ochratoxin A detection. Trac-Trends Anal. Chem..

[6] Duarte S.C., Lino C.M., Pena A. (2012). Food safety implications of ochratoxin A in animal-derived food products. Vet. J..

[7] Zhang G., Zhu C., Huang Y., Yan J., Chen A. (2018). A Lateral Flow Strip Based Aptasensor for Detection of Ochratoxin A in Corn Samples. Molecules.

[8] Zou X., Chen C., Huang X., Chen X., Wang L., Xiong Y. (2016). Phage-free peptide ELISA for ochratoxin A detection based on biotinylated mimotope as a competing antigen. Talanta.

[9] Kong D., Liu L., Song S., Suryoprabowo S., Li A., Kuang H., Wang L., Xu C. (2016). A gold nanoparticle-based semi-quantitative and quantitative ultrasensitive paper sensor for the detection of twenty mycotoxins. Nanoscale.

[10] Guan B., Zhang X. (2020). Aptamers as Versatile Ligands for Biomedical and Pharmaceutical Applications. Int. J. Nanomed..

[11] Song S.-H., Gao Z.-F., Guo X., Chen G.-H. (2019). Aptamer-Based Detection Methodology Studies in Food Safety. Food Anal. Methods.

[12] Nekrasov N., Jaric S., Kireev D., Emelianov A.V., Orlov A.V., Gadjanski I., Nikitin P.I., Akinwande D., Bobrinetskiy I. (2022). Real-time detection of ochratoxin A in wine through insight of aptamer conformation in conjunction with graphene field-effect transistor. Biosens. Bioelectron..

[13] Zhou W., Kong W., Dou X., Zhao M., Ouyang Z., Yang M. (2016). An aptamer based lateral flow strip for on-site rapid detection of ochratoxin A in Astragalus membranaceus. J. Chromatogr. B.

[14] Wang L., Ma W., Chen W., Liu L., Ma W., Zhu Y., Xu L., Kuang H., Xu C. (2011). An aptamer-based chromatographic strip assay for sensitive toxin semi-quantitative detection. Biosens. Bioelectron..

[15] Huang X., Aguilar Z.P., Xu H., Lai W., Xiong Y. (2016). Membrane-based lateral flow immunochromatographic strip with nanoparticles as reporters for detection: A review. Biosens. Bioelectron..

[16] Zhang R.L., Gao B., Zhang J., Cui H.Z., Li D.W. (2015). Propagation of PAMAM dendrimers on the carbon fiber surface by in situ polymerization: A novel methodology for fiber/matrix composites. Appl. Surf. Sci..

[17] Elancheziyan M., Senthilkumar S. (2019). Covalent immobilization and enhanced electrical wiring of hemoglobin using gold nanoparticles encapsulated PAMAM dendrimer for electrochemical sensing of hydrogen peroxide. Appl. Surf. Sci..

[18] Shen G., Xu H., Gurung A.S., Yang Y., Liu G. (2013). Lateral flow immunoassay with the signal enhanced by gold nanoparticle aggregates based on polyamidoamine dendrimer. Anal. Sci..

[19] Peng X.Y., Kang L.C., Pang F.Q., Li H.M., Luo R.F., Luo X.L., Sun F.X. (2018). A signal-enhanced lateral flow strip biosensor for ultrasensitive and on-site detection of bisphenol A. Food Agric. Immunol..

[20] Khan A.K., Rashid R., Murtaza G., Zahra A. (2014). Gold Nanoparticles: Synthesis and Applications in Drug Delivery. Trop. J. Pharm. Res..

[21] Lu S.Y., Lin C., Li Y.S., Zhou Y., Meng X.M., Yu S.Y., Li Z.H., Li L., Ren H.L., Liu Z.S. (2012). A screening lateral flow immunochromatographic assay for on-site detection of okadaic acid in shellfish products. Anal. Biochem..

[22] Anfossi L., Giovannoli C., Giraudi G., Biagioli F., Passini C., Baggiani C. (2012). A Lateral Flow Immunoassay for the Rapid Detection of Ochratoxin A in Wine and Grape Must. J. Agric. Food Chem..

[23] Anfossi L., Di Nardo F., Giovannoli C., Passini C., Baggiani C. (2013). Increased sensitivity of lateral flow immunoassay for ochratoxin A through silver enhancement. Anal. Bioanal. Chem..

[24] Zhou J.M., Yang Q.B., Liang C., Chen Y.M., Zhang X.L., Liu Z.X., Wang A.P. (2021). Detection of ochratoxin A by quantum dots-based fluorescent immunochromatographic assay. Anal. Bioanal. Chem..

[25] Duan H., Huang X.L., Shao Y.N., Zheng L.Y., Guo L., Xiong Y.H. (2017). Size-Dependent Immunochromatographic Assay with Quantum Dot Nanobeads for Sensitive and Quantitative Detection of Ochratoxin A in Corn. Anal. Biochem..

[26] Hao L.W., Chen J., Chen X.R., Ma T.T., Cai X.X., Duan H., Leng Y.K., Huang X.L., Xiong Y.H. (2021). A novel magneto-gold nanohybrid-enhanced lateral flow immunoassay for ultrasensitive and rapid detection of ochratoxin A in grape juice. Food Chem..

